# Preliminary Study of the Cancer Stem Cells’ Biomarker CD147 in Leukoplakia: Dysplasia and Squamous Cell Carcinoma of Oral Epithelial Origin

**DOI:** 10.7759/cureus.38807

**Published:** 2023-05-09

**Authors:** Vasileios Zisis, Dimitrios Andreadis, Pinelopi Anastasiadou, Konstantinos Vahtsevanos, Meni Akrivou, Ioannis S Vizirianakis, Athanasios Poulopoulos

**Affiliations:** 1 Oral Medicine/Pathology, Aristotle University of Thessaloniki, Thessaloniki, GRC; 2 Oral and Maxillofacial Surgery, Aristotle University of Thessaloniki, Thessaloniki, GRC; 3 Pharmacology, Aristotle University of Thessaloniki, Thessaloniki, GRC

**Keywords:** rt-qpcr, cd147, immunohistochemistry staining, oral leukoplakia, oral cancers, oscc, cancer stem cells

## Abstract

Objectives

Cancer stem cells (CSCs) are responsible for initiating the process of carcinogenesis de novo, as well as through the transformation of oral potential malignant disorders (OPMDs) to oral squamous cell carcinoma (OSCC). The aim of our study was to detect the expression of stemness-type CSC marker CD147 in oral leukoplakias (OLs), the most common OPMD, and OSCCs as well.

Materials and methods

This study focuses on the semiquantitative immunohistochemical pattern of the expression of the CSC protein biomarker CD147 in paraffin-embedded samples of 20 OSCCs of different grades of differentiation and 30 cases of OLs without or with different grades of dysplasia, compared to the normal oral epithelium in terms of cells’ stain positivity. Statistical analysis was performed through Statistical Package for Social Sciences (SPSS) version 25.0 (IBM SPSS Statistics, Armonk, NY) with Pearson chi-square test, and the significance level was set at 0.05 (p=0.05). In addition, the study clarified the expression of the respective gene of *CD147* through quantitative polymerase chain (qPCR), in paraffin-embedded samples of the two extreme graduations: OLs of mildly dysplastic or non-dysplastic cases (n=10 cases) and OSCCs of moderately/poorly differentiated cases (n=17). Statistical analysis was then performed through SPSS version 25.0 with an independent paired t-test, and the significance level was set at 0.05 (p=0.05).

Results

The gene *CD147* was expressed in all cases, although no statistically significant correlations were established. Regarding its protein products, the characteristic membranous staining of CD147 was noticed in the majority of the samples, mostly in the basal and parabasal layers of the epithelium. CD147 was upregulated significantly in the moderately and severely dysplastic OLs than in the mildly dysplastic and non-dysplastic OLs (p=0.008). Also, CD147 was upregulated significantly in the mildly dysplastic and non-dysplastic OLs than in the normal oral epithelium (p=0.012).

Discussion

The characteristic expression of CD147 in OLs and OSCCs’ lesions suggests the presence of stemlike cancer cells, illustrating an underlying effect on the early stages of oral dysplasia, in the OL stage. The clinical application of CD147 as prognostic factor requires the experimental evaluation in larger number of samples.

Conclusion

Stem cells play an important role in the process of carcinogenesis. A major goal in cancer research is the identification of specific biomarkers for the detection of cancer stem cells. CD147 is considered as an innovative stem cell marker. Our findings in oral mucosal potentially malignant disorders showed that CD147 is expressed more intensely in parallel with the progression of the grade of dysplasia in OL. On the other hand, in oral squamous cell carcinoma, CD147 expression remains stable regardless of the degree of differentiation.

## Introduction

Oral leukoplakia (OL), a well-established oral potential malignant disorder (OPMD), may be divided into two groups, based on the binary taxonomy proposed by WHO (2005) [1]. The first group includes non-dysplastic leukoplakia and mildly dysplastic leukoplakia, whereas the second group includes moderately dysplastic leukoplakia and severely dysplastic leukoplakia (binary taxonomy) (WHO, 2005) [2,3]. Oral squamous cell carcinoma (OSCC) is divided into three categories: well-differentiated, moderately differentiated, and poorly differentiated. Well-differentiated carcinoma consists of dermis-infiltrating strands of neoplastic cells with a detectable propensity toward keratinization (keratin spheres). The poorly differentiated end consists of cells that resemble primordial undifferentiated cells with a high degree of variety (WHO classification, 2017) [4].

Cancer stem cells (CSCs) represent a small subset of cancer cells and manifest self-renewal and the ability to differentiate into cancer cells. They are responsible for the phenomena of cancer invasion and metastasis, as well as the development of resistance to chemotherapy and radiotherapy. Targeting them successfully is therefore an important therapeutic goal and may be achieved by identifying specific antigens that are expressed by them [5]. Additionally, cancer stem cells are present both in potentially malignant lesions and in cancerous lesions, most probably intervening and mediating in the process of carcinogenesis [6].

CD147 or extracellular matrix metalloproteinase inducer (EMMPRIN) is a stemness cancer stem cell biomarker of somatic cancer cells and is indicative of the ability of the cell to remain in a stem cell state. CD147 is a glycosylated protein belonging to the immunoglobulin superfamily. CD147 presents in two forms: the transmembrane and soluble forms [7]. The transmembrane part consists of two segments, an extracellular domain and a cytoplasmic tail, that play an important role in the induction or stimulation of matrix metalloproteinase (MMP), while the soluble part has been shown to be a useful marker for hepatocellular carcinoma [7]. CD147 is present in epithelial cells, neuronal or nerve cells, myocardial cells, lymphoid cells, and germ cells and plays an important role in several biological processes, including fetal development, retinal function, nervous system development, and thymic T cell development [8]. CD147 is also expressed in a variety of cancers, such as head and neck squamous cell carcinomas, pancreatic adenocarcinomas, kidney chromophobic carcinomas, hepatocellular carcinomas, medullary breast adenocarcinomas, cervix carcinomas, and glioblastomas [8]. In OSCC cases, an association between increased CD147 expression and poor prognosis has been observed [8]. The dissolution of the extracellular matrix (ECM) is mediated by proteolytic enzymes, of which the most important category for the purpose of cancer invasion and metastasis is that of the matrix metalloproteinases (MMPs) [9]. The aim of this study was to examine the pattern of CD147 expression in OLs of all dysplastic cases and in cases of OSCCs of all differentiation grades.

## Materials and methods

Tissue samples

The paraffin-embedded tissue samples of OLs and OSCCs for both immunohistochemical and quantitative polymerase chain (qPCR) methods were obtained from the archives of the Department of Oral Medicine/Pathology, Dental School, Aristotle University of Thessaloniki (AUTh). Tissue specimens were derived from biopsies conducted in the period 2009-2019 as part of the routine diagnostic procedure in the Department of Oral Medicine/Pathology, School of Dentistry, Oral and Maxillofacial Surgery Clinic, “G. Papanikolaou” General Hospital, Aristotle University of Thessaloniki (AUTh) and the Oral and Maxillofacial Surgery Clinic of St. Luke Hospital, Thessaloniki, Greece. The tissues were fixed in a 10% formaldehyde solution to preserve their cellular structure and then were embedded into paraffin for long-term preservation.

The inclusion criteria were the highest feasible equitable involvement of males and females of comparable ages, behaviors (smoking and alcohol consumption), and medical history that did not influence the outcome of the experiment. The presence of enough precancerous or cancerous biological material (estimated as more than 70% per tissue specimen in order to avoid the possible positivity of non-epithelial cells for the relevant markers) in the paraffin cubes was an additional criterion for the procedure of qPCR particularly. The patients were informed and consented, and the whole study was approved by the Ethics Committee of the Dental School of Aristotle University of Thessaloniki, Greece (Nr 8/03.07.2019). Multiple 4 μm-thick sections for immunohistochemistry (IHC) and 10 μm-thick sections for qPCR were used (Jung Biocut 2035 Microtome, Leica Biosystems, Wetzlar, Germany).

Immunohistochemistry (IHC)

This study examined the immunohistochemical pattern of the expression of CD147 in tissue samples from cases of OLs of all degrees of dysplasia and OSCCs of all degrees of differentiation in comparison to cases of normal mucosa (healthy epithelium from reactive hyperplasias, e.g., fibroma). In detail, 30 cases of OL were chosen, which were further divided into two subgroups according to the WHO 2005 binary taxonomy for OLs. The first subgroup included 14 cases of non-dysplastic and mildly dysplastic OL, whereas the second subgroup included 16 cases of moderately and severely dysplastic OL.

Additionally, 21 cases of OSCCs were chosen, according to the WHO 2017 OSCC taxonomy based on the degree of differentiation, which were further divided into two subgroups. The first subgroup included 16 cases of moderately and poorly differentiated OSCC, whereas the second subgroup included five cases of well-differentiated OSCC. The control group consisted of five cases of normal oral epithelium adjacent to reactive hyperplasias (fibroma).

For the immunohistochemical technique application, the sections were mounted on slides (Polysine and Superfrost Plus, Thermo Scientific Menzel Gläser, Braunschweig, Germany), and the associated hematoxylin sections were examined for comparison. When applying the immunohistochemical technique to further process the sections, the anti-CD147 (sc-21746, Santa Cruz Biotechnology, Dallas, TX) was utilized at a dilution of 1:100 using the Dako EnVision FLEX+ system (Dako Denmark A/S, Glostrup, Denmark) as secondary stain detection system and the chromogenic agent application (DAB EnVision, Dako Denmark A/S, Glostrup, Denmark). The stepwise process included the antigen recovery (Dako EnVision FLEX+ Target Antigen Retrieval Solution, High pH, Dako Denmark A/S, Glostrup, Denmark), the application of primary antibody, the utilization of the Dako EnVision system’s secondary stain detection system, the chromogenic agent application (DAB EnVision, Dako Denmark A/S) (chromogen), and finally employing hematoxylin. The section was then affixed to the mounting plate and coated to protect and preserve the preparation over time. Subsequently, the sections were examined under the microscope to observe and record the results of the immunohistochemical staining evaluation. For microscopy and the recording of results, an Olympus CX31 microscope (Olympus LS, Tokyo, Japan) and an Olympus SC30 camera (Olympus Soft Imaging Solutions, Muenster, Germany) were utilized. The evaluation of the membranous staining of CD147 is obtained as a histochemical score by calculating the percentage of positive cells into a scale of 1-3 (positive cells: Ι, 6%-35%; ΙΙ, 36%-70%; and ΙΙΙ, 71%-100%) [10]. Especially, in OLs, the three-tier scale was defined as follows: Ι, the presence of positive cells in one-third of the epithelium; ΙΙ, the presence of positive cells in two-thirds of the epithelium; and ΙΙΙ, the presence of positive cells throughout the epithelium. Negative staining was defined as the presence of less than 5% of positively stained cells. The staining was considered to be positive when the cytoplasm, membrane, or nucleus was colored brown. Statistical analysis was performed through the Statistical Package for Social Sciences (SPSS) version 25.0 (IBM SPSS Statistics, Armonk, NY) with Pearson chi-square test and Fisher’s exact test depending on the sample size, and the significance level was set at 0.05 (p=0.05).

Quantitative polymerase chain reaction (qPCR)

In this study, the relative expression of the *CD147* gene (Custom CD147 Eurofins MWG, Qiagen, Hilden, Germany) through qPCR was examined in tissue samples of mildly dysplastic and non-dysplastic OL and moderately and poorly differentiated OSCC. Following the binary taxonomy of the WHO, 10 cases of non-dysplastic/mildly dysplastic OLs were chosen compared to 17 cases of moderately/poorly differentiated OSCC. Through this comparison, the two limits of the grading panel of premalignant and malignant lesions were evaluated for the stem cell marker CD147, indicating the significance of gene expression and in order to confirm the consequent presence of protein products.

After the deparaffinization of formalin-fixed paraffin-embedded tissues, using a deparaffinization solution (Qiagen Deparaffinization Solution {number 73504}, Qiagen, Hilden, Germany), RNA extraction was accomplished using the RNeasy FFPE Kit (Qiagen, Hilden, Germany) according to the manufacturer’s recommendations. DNase treatment was also performed to eliminate genomic DNA. To evaluate the quality and quantity of extracted RNA, spectrophotometry was conducted. Extracted RNA was prone to synthesize complementary DNA (cDNA) using Qiagen kit according to the manufacturer’s protocol. Fifty nanograms of total RNA was used as template to generate first-strand cDNA using a cDNA kit (Qiagen QuantiTect Rev. Transcription Kit {number 205311}, Qiagen, Hilden, Germany). Polymerase chain reaction (PCR) was carried out on 7500 Real-Time PCR System (Applied Biosystems, Foster City, CA) using KAPA SYBR FAST qPCR Master Mix kit (number KK4602) (Kapa Biosystems, Wilmington, MA) in a total volume of 10 μl with the following thermal cycling parameters: 95°C for three minutes, followed by 40 cycles of denaturation at 95°C for three seconds and annealing/extension at 60°C for 60 seconds. All reactions were carried out in triplicates, and the resulting text file was exported to Microsoft Excel (Microsoft® Corp., Redmond, WA). The expression of the gene was normalized to the average Ct value of β-actin (Qiagen QuantiTect Primer Assay (200) Hs_ACTB_1_SG QuantiTect Primer Assay, Qiagen, Hilden, Germany). The DNA sequence (5’-3’) of the primer for CD147 is F:CCATGCTGGTCTGCAAGTCAG and R:CCGTTCATGAGGGCCTTGTC and F:TTGCTGACAGGATGCAGAAG and R:TGATCCACATCTGCTGGAAG for the primer for β-actin. The mean expression of the gene under investigation was normalized by comparison to the mean expression of β-actin. In particular, ΔCt was calculated (Ctmean of CD147 minus the Ctmean of β-actin). The cases were divided into two subcategories: mildly dysplastic and non-dysplastic OL and moderately and poorly differentiated OSCC. Statistical analysis was performed through the Statistical Package for Social Sciences (SPSS) version 25.0 (IBM SPSS Statistics, Armonk, NY) with independent paired t-test, and the significance level was set at 0.05 (p=0.05).

## Results

Firstly, all the samples were positive (normal, OL, and OSCC), regarding the gene expression, in the qPCR, although no statistically significant correlations were established. The general observations that arose from the immunohistochemical staining were the following: CD147 staining was membranous and was noticed in the basal, parabasal, and prickle cell layers. CD147-positive cancer stem cells were noticed in the cancerous foci. Table 1 includes the localization and grade of the IHC’s normal, OL, and OSCC cases.

**Table 1:** Localization and grade of the IHC’s normal, OL, and OSCC cases. OL, oral leukoplakia; OSCC, oral squamous cell carcinoma; IHC, immunohistochemistry

Location	Normal (n=5)	Total OL (n=30)	Non-dysplastic and mildly dysplastic OL (n=14)	Moderately and severely dysplastic OL (n=16)	Total OSCC (n=20)	Well-differentiated OSCC (n=5)	Moderately and poorly differentiated OSCC (n=15)
Mucobuccal fold		2		2			
Tongue	3	18	7	11	13	3	10
Cheek	2	6	4	2	3		3
Palate		1		1			
Lip		1	1		2	2	
Corner of the mouth		1	1				
Gingiva		1	1				
Mouth floor					2		2

A summary of statistical analysis for the immunohistochemical staining score among OLs, OSCCs, and normal oral epithelium can be found in Table 2.

**Table 2:** IHC score of the IHC’s normal, OL and OSCC cases. OL, oral leukoplakia; OSCC, oral squamous cell carcinoma; IHC, immunohistochemistry

Biomarker	CD147			
IHC score	0	1	2	Total
Normal	2	3	0	5
Total OL	7	22	1	30
Non-dysplastic and mildly dysplastic OL	0	14	0	14
Moderately and severely dysplastic OL	7	8	1	16
Total OSCC	3	14	3	20
Well-differentiated OSCC	0	3	2	5
Moderately and poorly differentiated OSCC	3	11	1	15

Statistically significantly higher expression of CD147 was noticed in moderately and severely dysplastic leukoplakia (B1) than in mildly dysplastic and non-dysplastic leukoplakia (B2) (Pearson chi-square p-value=0.008). Statistically significantly higher expression of CD147 was noticed in non-dysplastic and mildly dysplastic leukoplakia (B2) than in normal oral epithelium (D) (Pearson chi-square p-value=0.012). In contrast, there was no statistically significant difference in the expression of CD147 between the moderately and severely dysplastic OLs, the well-differentiated OSCC, and the moderately and poorly differentiated OSCC. Overall, the levels of the expression of CD147, based on the aforementioned findings, are summarized in Table 3.

**Table 3:** Levels of the protein expression of CD147 in OL, OSCC, and normal oral epithelium. OSCC, oral squamous cell carcinoma; OL, oral leukoplakia

	Level of the expression of CD147
Poorly and moderately differentiated OSCC	++
Well-differentiated OSCC	++
Moderately and severely dysplastic OL	++
Mildly dysplastic and non-dysplastic OL	+
Normal oral epithelium	+

**Figure 1:**
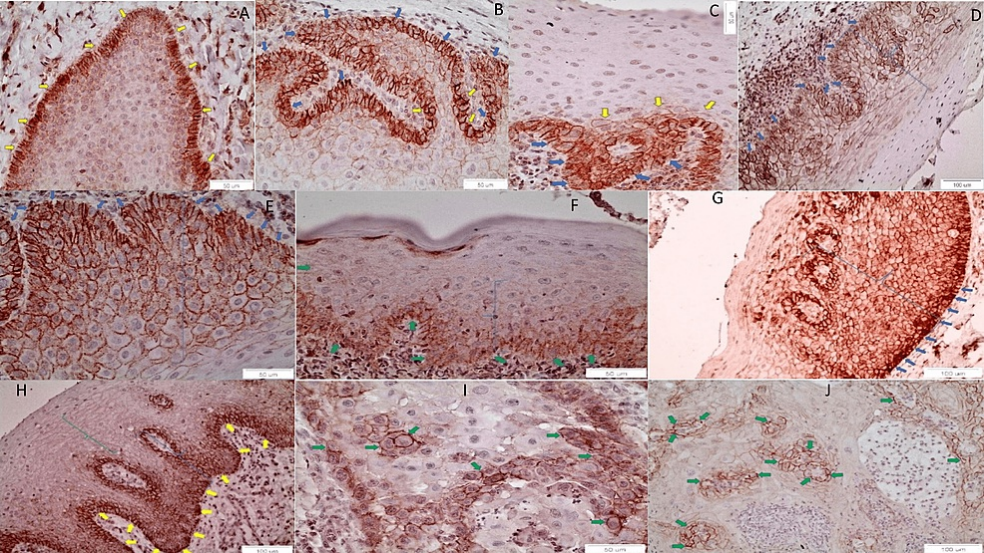
CD147 expression in normal oral epithelium, OLs, and OSCCs. OSCCs, oral squamous cell carcinomas; OLs, oral leukoplakias

In normal oral epithelium, CD147 revealed that a membranous staining was mildly expressed in the membrane of basal cells, characteristically in the interface area of the cells of the basal layer of the epithelial cells with the basal membrane (yellow arrows) (40×, Figure 1A). In a case of non-dysplastic OL, CD147 membranous staining was observed in the lower 1/3 of the epithelium, characteristically in the interface area of the cells of the basal layer of the epithelial cells with the basal membrane (the yellow arrows correspond to cells with more intense staining compared to the blue arrows) (40×, Figure 1B). In a case of mildly dysplastic OL, CD147 membranous staining was observed in the lower 1/3 of the epithelium, characteristically in the interface area of the cells of the basal layer of the epithelial cells with the basal membrane. The basal layer (blue arrows) was more intensely stained than the parabasal layer (yellow arrows) (40×, Figure 1C). In contrast, in moderately and severely dysplastic OL, the positive CD147 membranous staining was noticed in the lower 2/3 of the epithelium (Figure 1D, 1E, 1F). In particular, the most intense staining was present in the basal layer; the staining was less intense in the parabasal and the prickle layer, whereas the keratin layer was negative. In two cases of moderately dysplastic OL, the typical membranous staining of the lower 2/3 of the epithelium (blue bracket) was noticed with the characteristic membranous staining of the interface area between the basal epithelial layer and the basal membrane (blue arrows) (20×, Figure 1D; 40×, Figure 1E).

In a case of severely dysplastic OL, the typical membranous staining of the lower 1/2 of the epithelium (blue bracket) was noticed with the characteristic membranous staining of the interface area between the basal epithelial layer and the basal membrane (green arrows) (40×, Figure 1F). In a case of severely dysplastic epithelium, which is adjacent to well-differentiated OSCC, the membranous staining of CD147 is noticed, especially in the interface area between the basal epithelial layer and the basal membrane (blue arrows). The membranous staining is noticed in more than 2/3 of the epithelium (blue bracket), whereas the keratin layer was not stained (20×, Figure 1G). A similar pattern of the expression of CD147 is noticed in the severely dysplastic epithelium adjacent to moderately differentiated OSCC. Characteristic membranous staining of the interface area between the basal epithelial layer and the basal membrane is noticed (yellow arrows). Positive membranous staining is noticed in more than 2/3 of the epithelium (blue and green bracket), and a more intense staining is noticed in the blue bracket, which includes the basal and parabasal layers, than in the green bracket, which includes most of the prickle layer (20×, Figure 1H). Cancerous foci in a case of poorly differentiated OSCC, manifesting as poorly differentiated cells, was positively stained by CD147 (green arrows), in contrast to the neighboring cells, which are more differentiated (40×, Figure 1I). Cancerous foci, in a case of poorly differentiated OSCC, manifested as scattered, poorly differentiated, CD147 positively stained, cancer cells (green arrows) (20×, Figure 1J).

The epidemiological features of the tissue samples involved in the immunohistochemical experiment and the qPCR experiment are summarized in Table 4.

**Table 4:** Summary of the tissue samples used for the immunohistochemical experiment (marked as green) and the qPCR experiment (marked as yellow). OSCC, oral squamous cell carcinoma; OL, oral leukoplakia; qPCR, quantitative polymerase chain

	Diagnosis	Location	Gender	Age	Score
1	Non-dysplastic OL	Tongue	Female	45	1
2	Non-dysplastic OL	Tongue	Male	67	1
3	Non-dysplastic OL	Cheek	Female	60	1
4	Non-dysplastic OL	Tongue	Female	68	1
5	Non-dysplastic OL	Tongue	Male	69	1
6	Non-dysplastic OL	Tongue	Female	68	1
7	Non-dysplastic OL	Cheek	Female	58	1
8	Non-dysplastic OL	Cheek	Female	61	1
9	Mildly dysplastic OL	Tongue	Female	61	1
10	Mildly dysplastic OL	Lip	Female	38	1
11	Mildly dysplastic OL	Tongue	Male	46	1
12	Mildly dysplastic OL	Gingiva	Female	12	1
13	Mildly dysplastic OL	Cheek	Male	75	1
14	Mildly dysplastic OL	Mouth corner	Male	37	1
15	Moderately dysplastic OL	Tongue	Female	44	1
16	Moderately dysplastic OL	Tongue	Female	60	1
17	Moderately dysplastic OL	Tongue	Male	58	0
18	Moderately dysplastic OL	Tongue	Female	67	0
19	Moderately dysplastic OL	Tongue	Female	62	2
20	Moderately dysplastic OL	Cheek	Male	67	0
21	Moderately dysplastic OL	Tongue	Male	75	1
22	Severely dysplastic OL	Tongue	Male	43	0
23	Severely dysplastic OL	Mucobuccal fold	Female	75	0
24	Severely dysplastic OL	Tongue	Male	50	0
25	Severely dysplastic OL	Mucobuccal fold	Male	59	1
26	Severely dysplastic OL	Cheek	Male	66	1
27	Severely dysplastic OL	Tongue	Male	64	1
28	Severely dysplastic OL	Tongue	Male	45	0
29	Severely dysplastic OL	Palate	Male	72	1
30	Severely dysplastic OL	Tongue	Female	84	1
31	Well-differentiated OSCC	Lip	Male	58	1
32	Well-differentiated OSCC	Lip	Male	77	2
33	Well-differentiated OSCC	Tongue	Female	73	1
34	Well-differentiated OSCC	Tongue	Female	67	2
35	Well-differentiated OSCC	Tongue	Male	43	1
36	Moderately differentiated OSCC	Tongue	Female	47	2
37	Moderately differentiated OSCC	Tongue	Female	53	1
38	Moderately differentiated OSCC	Cheek	Male	45	1
39	Moderately differentiated OSCC	Cheek	Male	66	1
40	Moderately differentiated OSCC	Cheek	Male	61	1
41	Moderately differentiated OSCC	Mouth floor	Female	76	1
42	Moderately differentiated OSCC	Tongue	Male	75	1
43	Moderately differentiated OSCC	Tongue	Female	79	1
44	Moderately differentiated OSCC	Tongue	Female	72	1
45	Poorly differentiated OSCC	Tongue	Female	77	1
46	Poorly differentiated OSCC	Tongue	Male	52	0
47	Poorly differentiated OSCC	Tongue	Male	60	0
48	Poorly differentiated OSCC	Tongue	Female	77	1
49	Poorly differentiated OSCC	Tongue	Female	80	0
50	Poorly differentiated OSCC	Mouth floor	Female	82	1
51	Normal	Tongue	Female	49	1
52	Normal	Cheek	Male	81	0
53	Normal	Cheek	Female	59	0
54	Normal	Tongue	Male	69	1
55	Normal	Tongue	Female	72	1
56	Non-dysplastic OL	Mucobuccal fold	Male	66	-0.54
57	Non-dysplastic OL	Upper lip	Male	41	1.91167
58	Non-dysplastic OL	Lower lip	Female	63	-0.86
59	Non-dysplastic OL	Alveolar ridge	Female	34	1.095
60	Non-dysplastic OL	Cheek	Male	67	3.09
61	Mildly dysplastic OL	Tongue	Male	57	-0.985
62	Mildly dysplastic OL	Oral mucosa	Female	60	-3.265
63	Mildly dysplastic OL	Cheek	Male	69	0.655
64	Mildly dysplastic OL	Cheek	Male	42	3.16833
65	Mildly dysplastic OL	Mouth floor	Male	47	1.41
66	Moderately differentiated OSCC	Tongue	Male	51	-1.59
67	Moderately differentiated OSCC	Mandible	Female	59	-3.89
68	Moderately differentiated OSCC	Cheek	Male	63	-0.97833
69	Moderately differentiated OSCC	Cheek	Male	59	-3.42333
70	Moderately differentiated OSCC	Maxilla	Female	85	5.725
71	Moderately differentiated OSCC	Tongue	Female	63	9.255
72	Moderately differentiated OSCC	Tongue	Female	79	5.425
73	Moderately differentiated OSCC	Tongue	Female	86	4.955
74	Moderately differentiated OSCC	Mucobuccal fold	Female	77	1.59
75	Moderately differentiated OSCC	Mucobuccal fold	Female	75	7.52
76	Moderately differentiated OSCC	Tongue	Male	67	2.45
77	Moderately differentiated OSCC	Alveolar ridge	Male	72	-3.21667
78	Moderately differentiated OSCC	Retromolar fossa	Male	30	2.26
79	Moderately differentiated OSCC	Mandible	Male	46	4.395
80	Poorly differentiated OSCC	Mouth floor	Female	82	4.55667
81	Poorly differentiated OSCC	Mandible	Male	80	-4.12
82	Poorly differentiated OSCC	Alveolar ridge	Male	35	5.45167

## Discussion

Cancer stem cells constitute a small subset of cancer cells and are capable of self-renewal and differentiation into cancer cells. They are responsible for the invasion and metastasis of cancer, as well as the development of chemotherapy and radiotherapy resistance. Targeting them successfully is therefore an important therapeutic goal and may be achieved by identifying specific biomarkers that are expressed by them [5]. The most frequently used CSC markers for head and neck squamous cell carcinoma are summarized in Table 5.

**Table 5:** Most frequently used CSC biomarkers for head and neck squamous cell carcinoma. CSC: cancer stem cell

Biomarker	Gene	Location	Reference
CD44	CD44	Membrane	[11,12]
CD24	CD24	Cellular vesicles	[13]
CD98	SLC7A5-SLC3A2	Nucleus, membrane, and cytoplasm	[14]
c-MET	MET	Membrane and cytoplasm	[15,16]
CD133	PROM1	Membrane and cytoplasm	[17]
CD166	ALCAM	Membrane and cytoplasm	[18]
Notch1	NOTCH1	Nucleus	[19]
ALDH1	ALDHA1	Cytoplasm	[20]
SOX2	SOX2	Nucleus	[21]
OCT4	POU5F1	Nucleus	[22]
Nanog	NANOG	Nucleus	[22]

Additionally, cancer stem cells are present both in potentially malignant lesions and in cancerous lesions, most probably intervening and mediating in the process of carcinogenesis [6]. Leukoplakia is by far (82%) the most common oral potential malignant disorder (OPMD) [23]. The research hypothesis is whether a positive correlation exists between the degree of the presence of cancer stem cells, the degree of the presence of cancer stem cells’ biomarkers, the degree of dysplasia of oral leukoplakia, and the degree of the differentiation of oral squamous cell carcinoma [24,25]. When it was discovered that the sites of chronic inflammation gave rise to tumors and that inflammatory cells were present in tumor tissues in the 19th century, the link between inflammation and cancer was first established [26]. Extracellular matrix (ECM) of the tumor microenvironment, which connects tumor cells to nonmalignant stromal cells, enables the tumor to initially invade locally before metastasizing [27]. Collagen, fibronectin, elastin, and proteoglycans make up the ECM. During organogenesis, inflammation, and wound healing, the ECM is broken down, reconfigured, and reorganized [28]. ECM breakdown is mediated by proteolytic enzymes, of which the matrix metalloproteinase (MMP) group of enzymes is most crucial for tumor invasion and metastasis [9].

MMPs are zinc-dependent endopeptidases that promote tumor invasion and control angiogenesis related to tumors. They are divided into four main groups of MMPs, which are the collagenases, gelatinases, stromelysins, and membrane-type MMPs, based on a combination of amino acid sequence, peptide domain structure, and substrate specificity [29,30]. MMPs focus on the tumor cells’ membranous protrusions (invadopodia), which allow for cell invasion into nearby stroma, access to the vasculature, and ultimately metastasis [7]. MMP-7 expression is linked to metastases and a poor prognosis in OSCC cases [31], and MMP-9 is linked to decreased survival rate [32]. By lowering the expression of MMP-2 and MMP-9, curcumin treatment prevents OSCC from becoming invasive [33]. The increased expression of MMP-1, MMP-9, and CD147, an MMP activator, may be the cause of the intrabony OSCC invasion [34]. Additionally, CD147 promotes the production of MMPs and takes part in the invasion of tumor cells [34]. Bone invasion is associated with MMP and CD147 [35]. The ability of tumor cells, undergoing epithelial to mesenchymal transition, to infiltrate the surrounding stroma is caused by the CD147 activation of MMPs in the tumor microenvironment [36].

In order to target angiogenesis, a combination therapy involving an antiangiogenic drug and an anti-CD147 may be beneficial. CD147 functions as a co-receptor for vascular endothelial growth factor (VEGF) [37]. Through its effects on glucose metabolism and the inhibition of the p53 pathway, CD147 contributes to the metabolism and proliferation of cancer cells [38]. The CSC biomarker CD147 revealed a typical membrane staining, which was especially noticed in the cells of the basal, parabasal, and prickle layers. The consistent expression of CD147 across dysplasia and carcinoma may be justified by the expected expression of CD147 in the basal cell layer by normal stem cells, due to the self-renewal process. Our findings (higher levels of CD147 expression in OSCC cases and moderately and severely dysplastic leukoplakia than in mildly and non-dysplastic leukoplakia) are in accordance to the relevant bibliography (with the exception of one study, which is of Ueda et al. {2007} [39]), where it was reported that the more CD147 is expressed, the worse the clinical appearance is. This finding is reported in a variety of cancers located at the esophagus, salivary glands, anus, breast, liver, ovaries, endometrium, bone [40], and the urinary bladder [41].

Poor prognosis was associated to high CD147 expression in cases of OSCC [8]. CD147 is a co-receptor of the vascular endothelial growth factor (VEGF), meaning that a therapeutic regimen targeting CD147 may inhibit the phenomenon of neoangiogenesis, which is a necessary prerequisite for carcinogenesis [37]. CD147 influences also the metabolism and proliferation of cancer cells by affecting the glucose metabolism and inhibiting the p53 signaling pathway [38]. In the oral cavity OSCC, higher expression of CD147 is linked to higher induction of MMPs in the tumor microenvironment, enabling the invasion of cancer cells (after their epithelial to mesenchymal transition takes place) in the peritumoral stroma [36]. Regarding oral verrucous carcinoma (OVC), a noninvasive subtype of oral cancer, the evidence-based results are contradicting so far [42]. In one study, it was reported that ALDH1 and Notch1 were uncommonly detected in verrucous carcinoma [43], whereas in another, the significantly increased expression of ALDH1 and SOX2 is associated with verrucous carcinoma [42]. Another entity is the microinvasive or superficially invasive squamous cell carcinoma (SISCC). SISCC originates from a preexisting severe dysplasia or carcinoma in situ and is loosely defined as a horizontally and minimally vertically spreading squamous cell carcinoma with focal and/or superficial invasion of less than 2 mm and no deeper than the lamina propria [44,45]. The presence of cancer stem cells may initiate the breach of the basal membrane, which leads to the development of SISCC.

Further studies are required to identify the molecules responsible for these phenomena and epiphenomena. The innovative element in our study (statistical analysis not only between leukoplakia and OSCC in general but also between their subcategories) allowed for the conclusion to be drawn; that is, CD147 is expressed statistically significantly more when the degree of dysplasia in leukoplakia reaches the threshold of moderate to severe dysplasia. When this threshold is surpassed, the expression of CD147 remains relatively stable. This elevated expression may be attributed to the presence of cancer stem cells. This finding suggests that CD147 immunohistochemical staining may be useful for predicting which leukoplakias, especially the mildly dysplastic and the non-dysplastic ones, may become further dysplastic in the future. CD147 immunohistochemical staining was proven to be insufficient in distinguishing poorly and moderately differentiated cancers from the well-differentiated ones. CD147 is also expressed in normal oral epithelium; thus, the comparison of CD147 expression between OL and OSCC to normal oral epithelium is necessary in order to calibrate the results. The qPCR experiment showed that all of our samples were positive regarding the presence of CD147 but failed to establish any statistically significant correlation. There is not any bibliography on qPCR implementation in OL and OSCC cases for the study of CD147. The only reference on qPCR and cancer stem cells reported that the embryonic stem cells’ markers Nanog and OCT4 were overexpressed in cancers with lymph node metastasis than in cancers without lymph node metastasis [46]. The limitations of our study included the lack of follow-ups of the patients from whom the tissue specimens were derived, the lack of tumor-node-metastasis (TNM) classification and of the human papillomavirus (HPV) status regarding the OSCC included, and the lack of study of additional target molecules such as VEGF.

## Conclusions

Stem cells play an important role in the process of carcinogenesis. A major goal in cancer research is the identification of specific biomarkers for the detection of cancer stem cells. CD147 is considered an innovative stem cell marker. Our findings in oral mucosal potentially malignant disorders showed that CD147 is expressed more intensely in parallel with the progression of the grade of dysplasia in OL. On the other hand, in oral squamous cell carcinoma, CD147 expression remains stable regardless of the degree of differentiation. The future perspectives could be the study of more biomarkers in even larger samples of patients, through immunohistochemistry and qPCR, in order to better illustrate the nature and function of cancer stem cells and the establishment of correlations with clinical parameters of OL and OSCC including medical history, environmental habits and behaviors, prognosis, TNM classification, and five-year survival rate.

## References

[1] Warnakulasuriya S, Reibel J, Bouquot J, Dabelsteen E (2008). Oral epithelial dysplasia classification systems: predictive value, utility, weaknesses and scope for improvement. J Oral Pathol Med.

[2] Brouns ER, Baart JA, Bloemena E, Karagozoglu H, van der Waal I (2013). The relevance of uniform reporting in oral leukoplakia: definition, certainty factor and staging based on experience with 275 patients. Med Oral Patol Oral Cir Bucal.

[3] Kujan O, Oliver RJ, Khattab A, Roberts SA, Thakker N, Sloan P (2006). Evaluation of a new binary system of grading oral epithelial dysplasia for prediction of malignant transformation. Oral Oncol.

[4] Almangush A, Mäkitie AA, Triantafyllou A (2020). Staging and grading of oral squamous cell carcinoma: an update. Oral Oncol.

[5] Zisis V, Venou M, Poulopoulos A, Dimitrios A (2021). Cancer stem cells in head and neck squamous cell carcinoma: treatment modalities. Balk J Dent Med.

[6] Surendran S, Siddappa G, Mohan A (2017). Cancer stem cell and its niche in malignant progression of oral potentially malignant disorders. Oral Oncol.

[7] Nasry WH, Rodriguez-Lecompte JC, Martin CK (2018). Role of COX-2/PGE2 mediated inflammation in oral squamous cell carcinoma. Cancers (Basel).

[8] Monteiro LS, Delgado ML, Ricardo S (2014). EMMPRIN expression in oral squamous cell carcinomas: correlation with tumor proliferation and patient survival. Biomed Res Int.

[9] Curran S, Murray GI (2000). Matrix metalloproteinases: molecular aspects of their roles in tumour invasion and metastasis. Eur J Cancer.

[10] Kyrodimou M, Andreadis D, Drougou A (2014). Desmoglein-3/γ-catenin and E-cadherin/ß-catenin differential expression in oral leukoplakia and squamous cell carcinoma. Clin Oral Investig.

[11] Kokko LL, Hurme S, Maula SM, Alanen K, Grénman R, Kinnunen I, Ventelä S (2011). Significance of site-specific prognosis of cancer stem cell marker CD44 in head and neck squamous-cell carcinoma. Oral Oncol.

[12] Judd NP, Winkler AE, Murillo-Sauca O (2012). ERK1/2 regulation of CD44 modulates oral cancer aggressiveness. Cancer Res.

[13] Zimmerer RM, Ludwig N, Kampmann A (2017). CD24+ tumor-initiating cells from oral squamous cell carcinoma induce initial angiogenesis in vivo. Microvasc Res.

[14] Martens-de Kemp SR, Brink A, Stigter-van Walsum M (2013). CD98 marks a subpopulation of head and neck squamous cell carcinoma cells with stem cell properties. Stem Cell Res.

[15] Sun S, Wang Z (2011). Head neck squamous cell carcinoma c-Met⁺ cells display cancer stem cell properties and are responsible for cisplatin-resistance and metastasis. Int J Cancer.

[16] Lim YC, Kang HJ, Moon JH (2014). C-Met pathway promotes self-renewal and tumorigenecity of head and neck squamous cell carcinoma stem-like cell. Oral Oncol.

[17] Zhang Q, Shi S, Yen Y, Brown J, Ta JQ, Le AD (2010). A subpopulation of CD133(+) cancer stem-like cells characterized in human oral squamous cell carcinoma confer resistance to chemotherapy. Cancer Lett.

[18] Yan M, Yang X, Wang L (2013). Plasma membrane proteomics of tumor spheres identify CD166 as a novel marker for cancer stem-like cells in head and neck squamous cell carcinoma. Mol Cell Proteomics.

[19] Lee SH, Do SI, Lee HJ, Kang HJ, Koo BS, Lim YC (2016). Notch1 signaling contributes to stemness in head and neck squamous cell carcinoma. Lab Invest.

[20] Zhou C, Sun B (2014). The prognostic role of the cancer stem cell marker aldehyde dehydrogenase 1 in head and neck squamous cell carcinomas: a meta-analysis. Oral Oncol.

[21] Chou MY, Hu FW, Yu CH, Yu CC (2015). Sox2 expression involvement in the oncogenicity and radiochemoresistance of oral cancer stem cells. Oral Oncol.

[22] Chiou SH, Yu CC, Huang CY (2008). Positive correlations of Oct-4 and Nanog in oral cancer stem-like cells and high-grade oral squamous cell carcinoma. Clin Cancer Res.

[23] Pires FR, Barreto ME, Nunes JG, Carneiro NS, Azevedo AB, Dos Santos TC (2020). Oral potentially malignant disorders: clinical-pathological study of 684 cases diagnosed in a Brazilian population. Med Oral Patol Oral Cir Bucal.

[24] Saluja TS, Ali M, Mishra P, Kumar V, Singh SK (2019). Prognostic value of cancer stem cell markers in potentially malignant disorders of oral mucosa: a meta-analysis. Cancer Epidemiol Biomarkers Prev.

[25] Ortiz RC, Lopes NM, Amôr NG (2018). CD44 and ALDH1 immunoexpression as prognostic indicators of invasion and metastasis in oral squamous cell carcinoma. J Oral Pathol Med.

[26] Sarode SC, Sarode GS, Kalele K (2012). Oral lichenoid reaction: a review. Int J Oral Maxillofac Pathol.

[27] Coghlin C, Murray GI (2014). The role of gene regulatory networks in promoting cancer progression and metastasis. Future Oncol.

[28] Bonnans C, Chou J, Werb Z (2014). Remodelling the extracellular matrix in development and disease. Nat Rev Mol Cell Biol.

[29] Brown GT, Murray GI (2015). Current mechanistic insights into the roles of matrix metalloproteinases in tumour invasion and metastasis. J Pathol.

[30] Liotta LA, Kohn EC (2001). The microenvironment of the tumour-host interface. Nature.

[31] Mäkinen LK, Häyry V, Hagström J (2014). Matrix metalloproteinase-7 and matrix metalloproteinase-25 in oral tongue squamous cell carcinoma. Head Neck.

[32] Yu Q, Stamenkovic I (2000). Cell surface-localized matrix metalloproteinase-9 proteolytically activates TGF-β and promotes tumor invasion and angiogenesis. Genes Dev.

[33] Lee AY, Fan CC, Chen YA, Cheng CW, Sung YJ, Hsu CP, Kao TY (2015). Curcumin inhibits invasiveness and epithelial-mesenchymal transition in oral squamous cell carcinoma through reducing matrix metalloproteinase 2, 9 and modulating p53-E-cadherin pathway. Integr Cancer Ther.

[34] Erdem NF, Carlson ER, Gerard DA, Ichiki AT (2007). Characterization of 3 oral squamous cell carcinoma cell lines with different invasion and/or metastatic potentials. J Oral Maxillofac Surg.

[35] de Vicente JC, Fresno MF, Villalain L, Vega JA, Hernández Vallejo G (2005). Expression and clinical significance of matrix metalloproteinase-2 and matrix metalloproteinase-9 in oral squamous cell carcinoma. Oral Oncol.

[36] Papadimitropoulou A, Mamalaki A (2013). The glycosylated IgII extracellular domain of EMMPRIN is implicated in the induction of MMP-2. Mol Cell Biochem.

[37] Khayati F, Pérez-Cano L, Maouche K (2015). EMMPRIN/CD147 is a novel coreceptor of VEGFR-2 mediating its activation by VEGF. Oncotarget.

[38] Huang Q, Li J, Xing J (2014). CD147 promotes reprogramming of glucose metabolism and cell proliferation in HCC cells by inhibiting the p53-dependent signaling pathway. J Hepatol.

[39] Ueda K, Yamada K, Urashima M (2007). Association of extracellular matrix metalloproteinase inducer in endometrial carcinoma with patient outcomes and clinicopathogenesis using monoclonal antibody 12C3. Oncol Rep.

[40] Bovenzi CD, Hamilton J, Tassone P (2015). Prognostic indications of elevated MCT4 and CD147 across cancer types: a meta-analysis. Biomed Res Int.

[41] Peng J, Jiang H, Guo J, Huang J, Yuan Q, Xie J, Xiao K (2020). CD147 expression is associated with tumor proliferation in bladder cancer via GSDMD. Biomed Res Int.

[42] Percy Ida A, Prasanth T, Isaac Joseph T, Girish KL (2022). Evaluation and comparison of cancer stem cell markers aldehyde dehydrogenase1 (ALDH1) and sex determining region-Y Box2 (Sox2) in different grades of oral squamous cell carcinoma and oral verrucous carcinoma - an immunohistochemical study. EVALUATION AND COMPARISON OF CANCER STEM CELL MARKERS ALDEHYDE DEHYDROGENASE1 (ALDH1) AND SEX DETERMINING REGION-Y BOX2 (SOX2) IN DIFFERENT GRADES OF ORAL SQUAMOUS CELL CARCINOMA AND ORAL VERRUCOUS CARCINOMA: AN IMMUNOHISTOCHEMICAL STUDY.

[43] de Freitas Filho SA, Coutinho-Camillo CM, Oliveira KK, Bettim BB, Pinto CA, Kowalski LP, Oliveira DT (2021). Prognostic implications of ALDH1 and Notch1 in different subtypes of oral cancer. J Oncol.

[44] Haberland C, Sasaki C, Judson B, Virk R, Prasad M (2012). Superficially invasive squamous cell carcinoma of the oral cavity. Oral Surg Oral Med Oral Pathol Oral Radiol.

[45] Amit-Byatnal A, Natarajan J, Shenoy S, Kamath A, Hunter K, Radhakrishnan R (2015). A 3 dimensional assessment of the depth of tumor invasion in microinvasive tongue squamous cell carcinoma--a case series analysis. Med Oral Patol Oral Cir Bucal.

[46] Grubelnik G, Boštjančič E, Grošelj A, Zidar N (2020). Expression of NANOG and its regulation in oral squamous cell carcinoma. Biomed Res Int.

